# Role of autophagy and lysosomal drug sequestration in acquired resistance to doxorubicin in MCF-7 cells

**DOI:** 10.1186/s12885-016-2790-3

**Published:** 2016-09-29

**Authors:** Baoqing Guo, Adam Tam, Stacey A. Santi, Amadeo M. Parissenti

**Affiliations:** 1 Health Sciences North Research Institute, Sudbury, ON P3E 5J1 Canada; 2 Department of Biology, Laurentian University, Sudbury, ON P3E 2C6 Canada; 3 Division of Medical Sciences, Northern Ontario School of Medicine, Sudbury, ON P3E 2C6 Canada; 4 Faculty of Medicine, Division of Oncology, University of Ottawa, Ottawa, ON K1H 8M5 Canada

**Keywords:** Drug resistance, Doxorubicin, Autophagy, Drug sequestration, Lysosomes, Breast tumour cells

## Abstract

**Background:**

The roles and mechanisms involved in starvation-induced autophagy in mammalian cells have been extensively studied. However, less is known about the potential role for autophagy as a survival pathway in acquired drug resistance in cancer cells under nutrient-rich conditions.

**Methods:**

We selected MCF-7 breast tumor cells for survival in increasing concentrations of doxorubicin and assessed whether the acquisition of doxorubicin resistance was accompanied by changes in doxorubicin and lysosome localization and the activation of autophagy, as assessed by laser scanning confocal microscopy with or without immunohistochemical approaches. The ultrastructure of cells was also viewed using transmission electron microscopy. Cellular levels of autophagy and apoptosis-related proteins were assessed by immunoblotting techniques, while protein turnover was quantified using a flux assay.

**Results:**

As cells acquired resistance to doxorubicin, the subcellular location of the drug moved from the nucleus to the perinuclear region. The location of lysosomes and autophagosomes also changed from being equally distributed throughout the cytoplasm to co-localizing with doxorubicin in the perinuclear region. There was an apparent temporal correlation between the acquisition of doxorubicin resistance and autophagy induction, as measured by increases in monodansylcadaverine staining, LC3-II production, and co-localization of LAMP1 and LC3-II immunofluorescence. Electron microscopy revealed an increase in cytoplasmic vacuoles containing mitochondria and other cellular organelles, also suggestive of autophagy. Consistent with this view, a known autophagy inhibitor (chloroquine) was highly effective in restoring doxorubicin sensitivity in doxorubicin-resistant cells. Moreover, this induction of autophagy correlated temporally with increased expression of the selective cargo receptor p62, which facilitates the delivery of doxorubicin-damaged mitochondria and other organelles to autophagosomes. Finally, we suggest that autophagy associated with doxorubicin resistance may be distinct from classical starvation-induced autophagy, since Beclin 1 and Atg7 expression did not change upon acquisition of doxorubicin resistance, nor did recombinant Bcl2 overexpression or an Atg7 knockdown alter doxorubicin cytotoxicity.

**Conclusion:**

Taken together, our findings suggest that doxorubicin resistance in MCF-7 breast cancer cells is mediated, at least in part, by the activation of autophagy, which may be distinct from starvation-induced autophagy.

## Background

Autophagy is a normal physiological mechanism for cellular homeostasis, whereby damaged or defective cellular components, including mitochondria [1], the endoplasmic reticulum [2] and peroxisomes [3], are digested within the cell by fusion with lysosomes. A basal level of autophagy occurs in all cells to ensure that only functional organelles are retained [4]. Autophagy can also be induced by external stressors such as growth factor deprivation [5] or upon exposure to specific chemotherapy drugs [6–11]. The ultimate fate of cells under stress depends upon the net effect of apoptotic versus survival signals, often regulated by important cellular regulatory proteins such as Bcl2 and p53 [12, 13]. Under nutrient limiting conditions, autophagy permits cells to survive by metabolizing their own organelles as a source of energy. However, this survival mechanism is considered a “double-edged sword”, since cells can also die by prolonged autophagy (also referred to as type II programmed cell death) [14–16].

A number of investigations suggest that autophagy induction can promote resistance to cell death within tumor cells and has important implications for resistance to chemotherapy in cancer treatment [17]. For instance, up-regulation of autophagy by the drug rapamycin can protect several tumor cell lines from cell death through apoptosis [18]. In addition, the DNA damaging agents temozolomide and etoposide were found to induce an autophagy-associated increase in ATP production in multiple glioma cell lines, which protects the cells from death, possibly contributing to resistance to these drugs [19]. Activation of autophagy was also observed when growth factors were withdrawn in apoptosis-deficient cells [20]. It has also been suggested that autophagy induction may be associated with imatinib resistance in mouse lymphoid cells [11]. However, a definitive role for autophagy in acquired resistance to cytotoxic chemotherapy drugs, including a temporal association between acquired drug resistance and autophagy induction, has yet to be demonstrated.

It has been previously demonstrated that most of the weakly basic chemotherapeutic drugs, such as DNA-binding anthracyclines, can accumulate in lysosomes, especially in drug resistant cells [21–25]. Therefore, sequestration of chemotherapy drugs in lysosomes is widely considered to be a *bona fide* mechanism of resistance to weakly basic chemotherapy drugs in cancer cells. The use of lysosomotropic agents to restore the sensitivity of drug-resistant cells to chemotherapeutic drugs has been widely investigated [26, 27], as reviewed by Agostinelli [28] and Kaufmann [29]. As these agents inhibit vacuolar H^+^-ATPase [30] or change lysosomal membrane permeability [31–33], they would be expected to block the accumulation of weakly basic chemotherapy drugs in lysosomes. Lysosomotropic agents such as chloroquine have recently been shown to promote the ability of the chemotherapy drug paclitaxel to kill cancer stem cells through the inhibition of autophagic survival [34].

In this study, we investigated the role of autophagy and lysosomal drug sequestration in the acquisition of doxorubicin resistance in MCF-7 breast tumor cells. This involved the study of a panel of MCF-7 cells developed in our laboratory, whereby MCF-7 breast tumor cells were selected for survival in increasing concentrations of doxorubicin (MCF-7_DOX2_ cells). Aliquots of cells were retained at each doxorubicin dose elevation. These cells do not express several drug transporters associated with doxorubicin resistance in vitro, including Abcb1, Abcc2, or Abcg2. We did, however, observe elevated expression of the Abcc1 protein at the highest doxorubicin selection dose (dose 12) [35]. Using this panel of cell lines, we show in this study a strong temporal association between the acquisition of doxorubicin resistance and both the induction of autophagy and the sequestration of doxorubicin into lysosomes. We further provide evidence suggesting that the autophagy associated with doxorubicin resistance is distinct from starvation-induced autophagy. Blockage of this autophagy mechanism may represent a novel approach to cancer therapy, in particular for treatment of recurrent disease after prior chemotherapy administration.

## Methods

This study did not require ethics approval from an ethics review committee or board because the study did not involve animals, humans, human data, or material collected from humans or animals.

### Maintenance of MCF-7 cells and establishment of drug resistant variants

Human MCF-7 breast cancers cells (lot HTB-22, American Tissue Culture Collection) were grown in Dulbecco’s H21 medium (Princess Margaret Hospital, Toronto, ON) containing 10 % fetal bovine serum (FBS) (Hyclone), and incubated at 37 °C in a humidified 5 % CO_2_ atmosphere. Doxorubicin-resistant MCF-7_DOX2_ cells were generated in our laboratory by selecting MCF-7 cells for resistance to increasing concentrations (doses) of doxorubicin (PFS, USP, Pfizer), as described previously [35]. The passage numbers for the doxorubicin-resistant MCF-7 cell lines at selection doses 8 through 12 are 203, 216, 220, 227 and 257, respectively. As controls, parental MCF-7 cells were identically “selected” in the absence of drug to identical passage numbers. These cell lines are referred to as “co-cultured control cell lines” for the above selection doses and help to account for any changes in drug sensitivity or other cell phenotypes associated with increased passage in culture. All of the cells used in experiments were not subcultured for more than 10 passages after thawing from frozen stocks. The parental cell line (MCF-7) has been authenticated by the American Tissue Culture Collection and all cell lines are free of mycoplasma contamination.

### Measurement of cellular drug sensitivity

The sensitivity of cells to doxorubicin at various doxorubicin selection doses was measured using a clonogenic assay as described previously [36]. The concentration of doxorubicin at which the number of colonies formed in the assay was reduced by 50 % (the IC50) was computed for both MCF-7_CC_ and MCF-7_DOX2_ cells. The degree of drug resistance in MCF-7_DOX2_ cells (the resistance factor) was then determined by dividing the IC50 value for MCF-7_DOX2_ cells (at that selection dose) by the IC50 value for MCF-7_CC_ cells (at a similar passage number). As an example of cell nomenclature, MCF-7_DOX2–10_ and MCF-7_CC10_ cells represent cells selected in the presence of doxorubicin to dose level 10 and their co-cultured control cells at that selection dose, respectively.

### Localization of cellular organelles and proteins by confocal microscopy

The location of mitochondria, lysosomes, and autophagosomes (and their possible co-localization with doxorubicin) in MCF-7_CC10_ and MCF-7_DOX2–10_ cells were investigated using laser scanning confocal microscopy after staining with MitoTracker® (red fluorescence) or LysoTracker® (green fluorescence), both from Molecular Probes, Thermo Fisher Scientific), or monodansyl cadeverine (MDC) from Sigma-Aldrich Chemicals, respectively. All images were obtained by confocal miscroscopy (model 510 Meta, Carl Zeiss, Toronto, ON) using lasers at specific wavelengths or under UV light. The location of doxorubicin was also visualized through confocal microscopy, since the drug naturally has red fluorescence. For the above experiments, MCF-7_CC10_ and MCF-7_DOX2–10_ cells were grown on glass coverslips for 2 days in the absence of drug in a 6 cm tissue culture plate covered with tissue culture medium. The cells were then treated with 2 μM doxorubicin [alone or together with 10 μM chloroquine (Sigma-Aldrich Chemicals)] for 8 and 48 hours for MCF-7_CC10_ and MCF-7_DOX2–10_ cells, respectively. The concentration of doxorubicin chosen for these experiments, while much higher than necessary for cytotoxicity, was the minimum concentration that permitted reliable visualization of the drug’s location in cells by its fluorescence. After staining with 50 nM LysoTracker® (using the manufacturer’s protocol) or staining with 50 μM MDC for 10 min, the coverslips were washed briefly in PBS and mounted on glass slides for examination by laser scanning confocal microscopy. All the images were taken using the same parameters for accurate comparison between treatments in one particular experiment, with the aid of an Argon laser excitation at 488 nm and emission at 560 nm (with a LP filter for doxorubicin fluorescence) and excitation at 458 nm and emission at 505–530 nm (with a BP filter for LysoTracker® fluorescence). Cells were stained with MDC after LysoTracker® staining. The filters for obtaining images by laser scanning microscope were set to Fset01 (blue), Fset17 (green) and Fset28 (red) and excited with UV light.

The locations of autophagosomes and lysosomes were also assessed by immunofluorescence using antibodies against LC3 (Cat# 2775, Cell Signaling) and LAMP1 (Cat# Sc-20011 H4A3, Santa Cruz), respectively, in an approach similar to that described by Nakagawa et al. [37]. According to the manufacturer, the former antibody preferentially binds to LC3 conjugated to phosphatidylethanolamine (LC3-II), which is recruited to autophagosomal membranes [38]. Hence, it is highly useful for visualizing the location of autophagosomes. Mitochondria were visualized by laser scanning confocal microscopy after staining with MitoTracker™ (Thermo Fisher). Both MCF-7_DOX2–10_ and MCF-7_CC10_ cells were grown on glass coverslips with or without prior treatment with 50 nM bafilomycin A1 for 24 h (to block the turnover of LC3-II, once formed). The cells on coverslips were fixed with 4 % formaldehyde in PBS. Immunocytochemical staining of cells with either anti-LC3 or anti-LAMP1 antibodies was performed as described by the manufacturer (Cell Signaling). Goat anti-rabbit IgG-TR (Cat# sc-2780, Santa Cruz) and goat anti-mouse IgG-FITC (Cat# sc-2010, Santa Cruz) secondary antibodies were used for detection of the LC3 or LAMP1 antibodies. To confirm the consistency of LC3 and LAMP1 staining, two fluorophores were switched between two secondary antibodies for the two color staining. For assessment of the location of mitochondria and autophagosomes, cells were labeled with 50 nM MitoTracker™ for 15 min prior to fixation. Immunostaining was then performed using the anti-LC3-II and goat anti-rabbit IgG-FITC antibodies. The settings for fluorescence detection were the same as described above. Images chosen were highly representative of cells views in a minimum of 5 fields (5–10 cells per field) from duplicate slides obtained in 2 to 3 independently performed experiments.

### Transmission electron microscopy

For visualization of cellular ultrastructure by electron microscopy, cells were grown in 10 cm petri dishes to about 70 % confluence, after which the cells were released by trypsin treatment, washed once with PBS, and harvested by centrifugation. The cell pellet was resuspended in 10 ml of ice cold 3 % glutaraldehyde fixative in 0.1 M sodium cacodylate buffer (pH 7.2) for 35 min at 4 °C. The cells were then collected by centrifugation and resuspended in 1 ml of ice cold 0.2 M sodium cacodylate buffer (pH 7.2). The samples were then sent to the University of Western Ontario with cold packs for embedding, sectioning, and visualization by electron microscopy.

### Immunoblotting experiments using whole cell lysates

Whole cell extracts of cells were prepared in modified RIPA buffer containing 1 % NP40, 0.5 % sodium deoxycholate, 1 % SDS, and 1 Complete™ protease inhibitor tablet for every 50 ml of buffer. Cultured cells were grown as a monolayer for 2 days until cell density reached 50–60 % confluence in 10 cm tissue culture plates. Twenty-four hours prior to protein extraction, the cells were treated with or without 50 nM bafilomycin A1 for 24 h under standard mammalian cell culture conditions. The culture medium was removed and the cells rinsed twice with PBS. To each plate, 0.7 ml of chilled modified RIPA buffer was added. The cells were scraped from the plates using a cell lifter, transferred to a 1.5 ml microfuge tube, and passed through a 21 gauge needle repeatedly to ensure efficient cell lysis and to shear any DNA present. The protein concentration for the whole cell extracts was determined using the BCA protein assay reagent kit (Pierce). Protein samples (30 μg) from whole cell extracts were used for SDS-PAGE analysis on 12 % or 10 % polyacrylamide gels based on the molecular weight of the target protein. Electrophoresis, protein transfer and immunoblotting were performed using standard procedures.

### Measurement of the degradation of long-lived proteins (flux assay)

The degradation of long-lived proteins was measured using a modification of the standard “flux assay” [39]. Cells were seeded in 6-well plates for 48 h in DMEM-H21 medium with 5 % FBS in a humidified incubator with 5 % CO_2_. When the cell density reached about 50–60 % confluence, the medium was replaced with fresh medium supplemented with 0.2 μCi/mL [14C (U)] L-valine (MC-277, Moravek Biochemicals) and incubated for 24 h at 37 °C. Unincorporated radioisotope was then removed by three PBS washes. Cells were then incubated with 10 mM unlabeled valine (Sigma-Aldrich) for 3 h to allow for short-lived protein turnover. The medium was then replaced with fresh medium containing 10 mM unlabeled valine in the absence or presence of 10 μM chloroquine or 1 μM rapamycin (R8781, Sigma-Aldrich) in order to inhibit or activate autophagy, respectively. After a 24 or 48 h incubation period, the medium was collected from the wells. The medium with some detached cells was mixed with BSA (5 mg/ml final concentration) and 10 % trichloroacetic acid (TCA; Sigma-Aldrich), after which proteins in the medium were allowed to precipitate at 4 °C for 30 min. The precipitated proteins (along with detached cells) were harvested by centrifugation at 600 × g for 10 min, leaving behind soluble radiolabeled proteins in the supernatant. The adherent cells remaining in the tissue culture flasks were also collected by scraping in 0.5 ml of 10 % TCA, after which proteins were allowed to precipitate at 4 °C for 30 min. The cells and precipitated proteins where then harvested by centrifugation at 600 × g for 10 min, again leaving behind soluble proteins in the supernatant. Fifty μl of the supernatant from cells and 222 μl of supernatant from the medium were combined and added to scintillation vials with 5 ml of scintillation fluid. This mixture represents the acid-soluble radioactivity from degraded proteins. The TCA-precipitated protein preparations from the detached cells in the medium and adherent cells were each solubilized in 500 μl of solubilization buffer (0.1 N NaOH + 0.1 % SDS). Fifty μl from each solubilized pellet was then added to scintillation vials with 5 ml of scintillation fluid. This mixture constituted the TCA-percipitable radioactivity from both detached and adherent cells. After allowing the vials to sit overnight at room temperature, the radioactivity in the vials was quantified by liquid scintillation counting (Beckman Coulter LS6500). The total protein degradation (% proteolysis) was measured by dividing the TCA-soluble radioactivity by the radioactivity in the precipitated proteins. For all experiments, values were reported as means ± S.D. (n = 3). Statistical differences between the two groups were determined by the Student’s *t*-test with Sigma plot 11.0 for Microsoft Windows. An identical experiment without isotope labeling was performed for protein extraction and immunoblot analysis of LC3-II expression after 48 h treatment.

### Inhibition of ATG7 expression by siRNA interference

MCF-7_CC10_ and MCF-7_DOX2–10_ cells were grown on 10 cm plates to 30–40 % confluence in antibiotic-free DMEM medium supplemented with 10 % FBS and left to adhere overnight. The next day, the culture media was removed and the cells were washed with PBS, after which 12 ml of Opti-MEM I media (Invitrogen) were added to each plate prior to cell transfection with Lipofectamine 2000 (Invitrogen), using the manufacturer’s instructions. Briefly, an ATG7-specific siRNA oligo (Ambion Silencer® Select) was added to 1.5 ml of Opti-MEM I medium at a 20 nM final concentration in 1 well of a 6-well plate. In a separate well, 30 μl of Lipofectamine 2000 was added to 1.5 ml of Opti-MEM I medium. After 5 min incubation, the two solutions were mixed (3 ml in total). An identical procedure was performed for a Silencer® Select negative control siRNA (Ambion). The mixture was incubated for 20 min, then added to cultures of the above cell lines. The plates were incubated at 37 °C for 24 h, after which the medium was removed and replaced with antibiotic-free DMEM medium, supplemented with 10 % FBS. At 48 h post-transfection, the cells were washed, trypsinized, counted, and plated for either a clonogenic assay or a flux assay (as described above). An aliquot of cells was retained from each transfection and proteins extracted (also as described above) in order to assess the efficiency of gene knockdown using immunoblotting experiments. The siRNA sequences used in the study are as follows: ATG7-1: 5′-GGAACACUGUAUAACACCAtt-3′ and 5′-UGGUGUUAUACAGUGUUCCaa-3′. ATG7-3: 5′-GAAGCUCCCAAGGACAUU-Att-3′ and 5′-UAAUGUCCUUGGGAGCUUCat-3′.

## Results

### Acquisition of resistance to doxorubicin in MCF-7 cells and restoration of sensitivity by chloroquine treatment

**Fig. 1:**
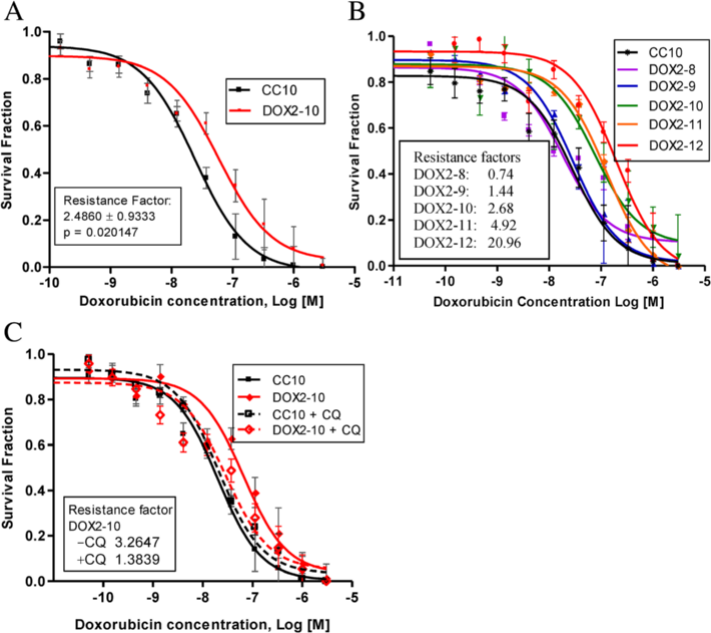
Doxorubicin sensitivity for various cell lines with or without the autophagy inhibitor chloroquine. The sensitivity of MCF-7_DOX2_ cells to doxorubicin was measured using a clonogenic assay. Cells were selected in increasing concentrations of doxorubicin to selection doses 7 (6.5 nM), 8 (19 nM), 9 (29 nM), 10 (44 nM), 11 (65 nM) and 12 (98.1 nM). The doxorubicin sensitivity of MCF-7 cells selected in the absence of doxorubicin to a passage number equal to dose level 10 (MCF-7_CC10_ cells) was also assessed (panels **a** and **b**). The sensitivity of the MCF-7_DOX2–10_ and MCF-7_CC10_ cell lines to doxorubicin in the absence or presence of chloroquine was also assessed (**c**). Chloroquine was dissolved in water, thus negating the need for a vehicle control in these experiments. Resistance factors represent the extent of resistance to doxorubicin, as calculated by dividing the IC_50_ of the drug-selected cell lines by the IC_50_ for its co-cultured control at the same passage number. The data points in Fig. 1a represent the average (± S. E.) of six independent experiments. The data points in Fig. 1b and c are representative of three independent experiments

Using clonogenic assays to measure drug sensitivity, we observed that resistance to doxorubicin in MCF-7 cells was acquired when the doxorubicin selection concentration reached or exceeded a specific ‘threshold’ dose, as we described previously [35]. The doxorubicin concentrations corresponding to the various selection doses (1 through 12) have been previously published [35]. Drug resistance was acquired at selection dose 9 (IC_50_ of 29 nmol/L; resistance factor 1.4; Fig. 1b). Cell lines were named to distinguish them from a prior round of selection (selection 2 in this case) and to reflect the maximum dose to which cells were selected. For example, MCF-7_DOX2_ cells selected in doxorubicin to dose level 10 were referred to as MCF-7_DOX2–10_ cells, while equivalent MCF-7_CC_ cells “selected” in the absence of drug to the same passage number were referred to as MCF-7_CC10_ cells. Strong resistance to doxorubicin (>2-fold) was only achieved when the doxorubicin selection dose reached 44 nmol/L (dose 10; IC_50_ of 75 nmol/L; resistance factor 2.5–2.7, as depicted in Figs. 1a and b. Resistance then progressively increased with increasing doxorubicin concentrations up to a maximally tolerated selection dose of 98 nmol/L at dose 12 (IC_50_ of 200 nmol/L; resistance factor 21). Data for selection doses 8 to 12 are depicted in Fig. 1b. Selection doses 8, 9, 10, 11, and 12 were 6.5, 19, 29, 44, 65, and 98 nM doxorubicin, respectively. “Co-cultured control” (MCF-7_CC_) cells exhibited little change in sensitivity to doxorubicin, despite long-term propagation in cell culture (data not shown).

To begin to assess whether autophagy might be involved in doxorubicin resistance, doxorubicin sensitivity was examined for MCF-7_CC_ and MCF-7_DOX2_ cells in the absence or presence of chloroquine---a compound known to inhibit autophagy by blocking the fusion of autophagosomes to lysosomes [40, 41]. As demonstrated in Fig. 1c, chloroquine treatment strongly increased sensitivity of MCF-7_DOX2–10_ cells to doxorubicin. In fact, sensitivity of MCF-7_DOX2–10_ cells to doxorubicin in the presence of chloroquine was almost equivalent to MCF-7_CC10_ cells. Interestingly, chloroquine had no effect on doxorubicin sensitivity in MCF-7_CC10_ cells (Fig. 1b). These findings suggest that acquisition of doxorubicin resistance at dose level 10 (44 nM selection dose) may be associated with induction of autophagy, since blockage of autophagy restored sensitivity to doxorubicin.

### Altered doxorubicin localization in MCF-7_DOX2–10_ cells

**Fig. 2:**
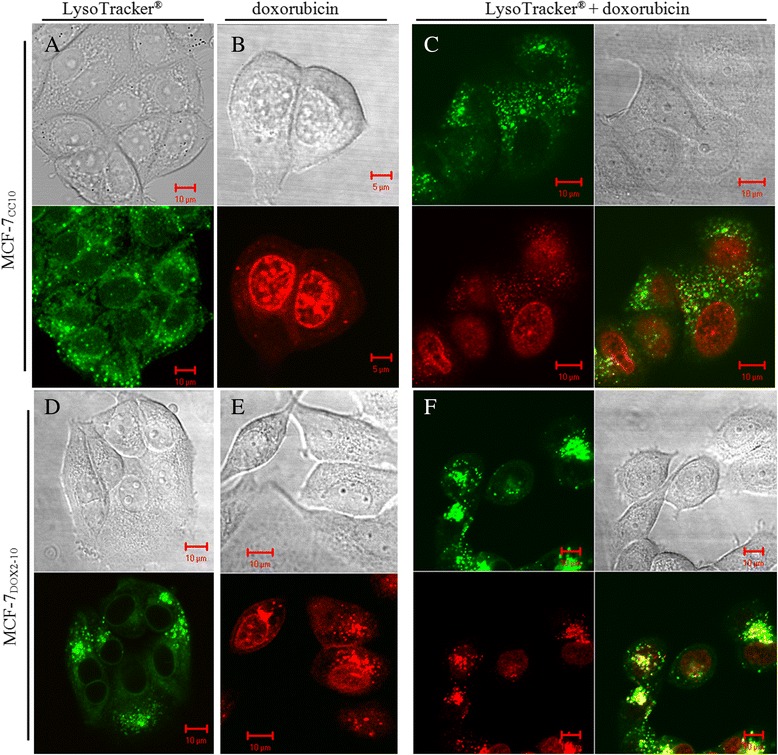
Distribution of lysosomes and doxorubicin in MCF-7_CC10_ and MCF-7_DOX2–10_ cells. Lysosomes in cells were visualized by laser scanning confocal microscopy after labelling with LysoTracker® and appeared green in colour. Doxorubicin distribution is depicted in red due to its autofluorescent nature. The images in this figure are representative of approximately 100 cells viewed on two separately stained slides in two independent experiments. Each image represents at least 10 microscopic photos taken from the two experiments. The localization of lysosomes and doxorubicin was also confirmed in three dimensions using stacked images obtained by confocal microscopy (data not shown) in two replicate experiments

Because of the autofluorescent property of doxorubicin, the distribution of this drug within cells could be observed using laser scanning confocal microscopy. We thus used this approach to visualize the location of doxorubicin within wildtype and doxorubicin-resistant cells. The drug was clearly localized predominantly within the nucleus of MCF-7_CC10_ cells, with some very minor punctate fluorescence within the cytoplasm (Fig. 2b). While single plane images of these cells suggest that most of doxorubicin is localized to the nuclear membrane, three dimensional views by stacking of the planar images indicated that the drug localized to regions within the nucleus (data not shown). A very small amount of drug appeared to be located on the nuclear membrane. In contrast, doxorubicin fluorescence was considerably reduced in MCF-7_DOX2–10_ cells, even after 48 h of incubation with the drug. The majority of the drug was localized to the perinuclear region in these cells (Fig. 2e). These findings suggest reduced doxorubicin accumulation into MCF-7_DOX2–10_ cells, of which the majority of the drug was not associated with its target (extranuclear). This could account, at least in part, for the observed resistance to doxorubicin. Subsequent drug uptake studies with radiolabelled doxorubicin confirmed the strongly reduced drug accumulation into MCF-7_DOX2–10_ cells relative to MCF-7_CC10_ cells (data not shown).

### Clustering of lysosomes upon selection for doxorubicin resistance

To identify the organelles within the perinuclear region to which doxorubicin may localize in MCF-7_DOX2_ cells, we visualized the location of lysosomes and mitochondria in MCF-7_CC10_ and MCF-7_DOX2–10_ cells using Lysotracker® and Mitotracker™, respectively. Interestingly, we observed that the distribution of lysosomes changed as cells developed resistance to doxorubicin. Lysosomes were evenly distributed throughout the cytoplasm in MCF-7_CC10_ cells (Fig. 2a). In contrast, these organelles were found to be clustered within the perinuclear region in MCF-7_DOX2–10_ cells, as indicated by intense punctate staining in a crescent shape towards one side of the nucleus (Fig. 2d). Moreover, unlike in MCF-7_CC10_ cells, the lysosomes in MCF-7_DOX2–10_ cells exhibited a very similar subcellular distribution pattern to that of doxorubicin, although some doxorubicin remained associated with the nucleus in MCF-7_DOX2–10_ cells (compare Fig. 2d and e).

### Co-localization of doxorubicin and Lysotracker® staining in MCF-7_DOX2–10_ cells

As both doxorubicin and Lysotracker® staining in MCF-7_DOX2–10_ cells appeared as clustered granules in the perinuclear region, we then assessed whether there was co-localization of doxorubicin and Lysotracker® staining by incubating MCF-7_CC10_ and MCF-7_DOX2–10_ cells with LysoTracker® after doxorubicin treatment. It was found that doxorubicin staining in MCF-7_DOX2–10_ cells co-localized for the most part with Lysotracker® staining, as visualized in the overlay images of green and red fluorescence exhibited in Fig. 2f. Some areas of clear green fluorescence in the overlay images (Fig. 2f) suggested that not all lysosomes contained doxorubicin. Addition of bafilomycin A1, a vacuolar H^+^-ATPase inhibitor that reduces vesicle acidification [42–44] almost completely eliminated the punctate lysosomal staining by LysoTracker® in MCF-7_DOX2–10_ cells (data not shown). In addition, the 30 min pre-incubation of these cells with bafilomycin A1 caused a complete loss of perinuclear doxorubicin accumulation, with no co-localization with lysosomes in the vast majority of cells (data not shown).

### Perinuclear lysosomes containing doxorubicin also exhibit positive monodansyl cadaverine staining

**Fig. 3:**
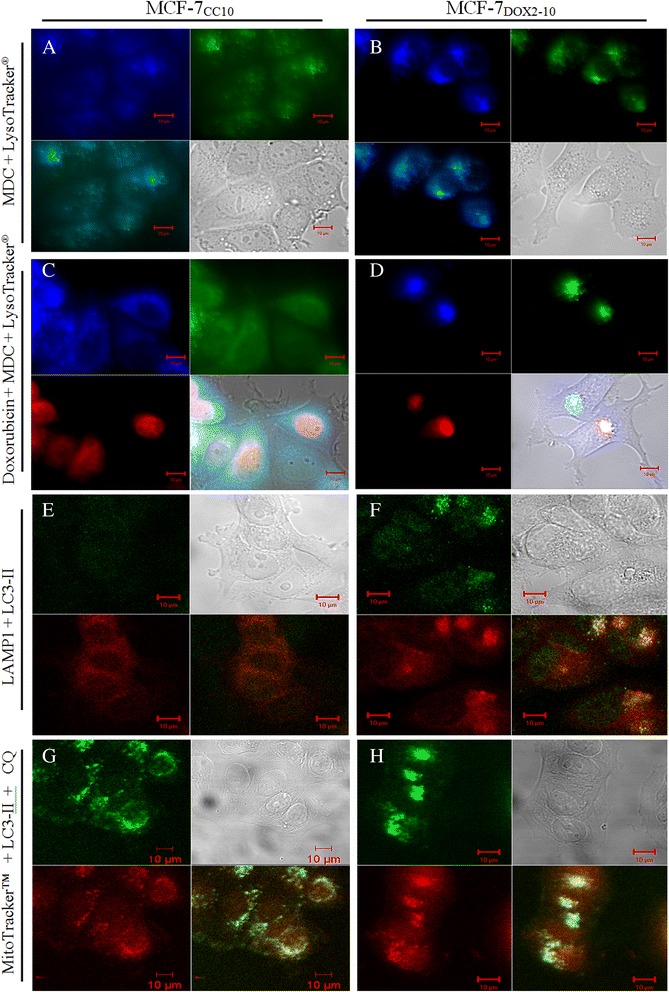
Laser scanning confocal microscope images of fluorescently labelled live MCF-7_CC10_ and MCF-7_DOX2–10_ cells (**a-d**) or identical cell lines fixed and immunohistochemically-stained with epitope-specific antibodies (**e-h**). Panels A through D represent cells stained with MDC (blue) and LysoTracker® (green) with or without doxorubicin treatment. In panels E and F, cells were fixed in formaldehyde and immunohistochemically stained with anti-LC3-II (green) and anti LAMP1 (red) antibodies. For panels G and H, cells were labelled with MitoTracker™ (red) and followed by formaldehyde fixation and immunocytochemical staining with an anti-LC3-II antibody (green) after 24 h treatment with 10 μM chloroquine (CQ) prior to labelling. The images in this figure are representatives of approximately 100 cells examined on two separately stained slides. Each image represents one of 10 microscopic photos taken from two independent experiments. The staining and phenotype consistency were also confirmed in at least two biological replicates by 3D image/video (data not shown)

Monodansyl cadaverine (MDC) is an autofluorescent dye shown empirically to localize to late autophagolysosomes but not endosomes in cells [45] This dye, when trapped in acidic and membrane-rich organelles, exhibits increased fluorescence. To provide evidence of a link between the acquisition of doxorubicin resistance and increased autophagy (which requires the formation of autophagolysosomes), we then compared the MDC staining of MCF-7_CC10_ and MCF-7_DOX2–10_ cells after incubation with doxorubicin, followed by Lysotracker® staining. Visualization of the blue MDC staining and the green Lysotracker® staining in MCF-7_DOX2–10_ cells revealed that, for the most part, there was a strong co-localization of the blue and green fluorescence, yielding autophagolysosomes exhibiting a bright blue hue (Fig. 3b). If cells were treated with red-fluorescing doxorubicin prior to staining with MDC and Lysotracker®, there was a strong co-localization of blue, green, and red fluorescence, yielding lysosomes of a bright violet color (Fig. 3d). These findings suggested that many of the lysosomes containing doxorubicin may also have been autophagolysosomes. Similar experiments in MCF-7_CC10_ cells revealed, not surprisingly, that there was some co-localized Lysotracker® and MDC staining (ie. some of the lysosomes were autophagolysosomes), but doxorubicin resided clearly in the nucleus of these cells (Figs. 3a and c). The partial co-localization of lysosomes with autophagosomes in doxorubicin-resistant cells was further confirmed by immunohistochemical approaches. Lysosomes and autophagosomes were visualized in MCF-7_DOX2–10_ cells by immunofluoresence with LAMP1 and LC3-II antibodies, respectively. As shown in Fig. 3f, a strong proportion of the red fluorescence generated by the LAMP antibody co-localized with the green fluorescence produced by the LC3-II antibody, producing a yellow hue. There was some red LAMP1-related fluorescence that did not co-localize with LC3-II fluorescence. This is understandable, since all lysosomes would not be expected to be autophagolysosomes. It is important to note that single staining with each primary antibody with an appropriate secondary antibody did not produce detectable “bleed-through” between the red, green, and blue regions of the fluorescent spectrum (data not shown).

Unlike MCF-7_DOX2–10_ cells, MCF-7_CC10_ cells showed very low levels of LC3-II and LAMP1-related immunofluorescence and this fluorescence was equally distributed throughout the cytoplasm (Fig. 3e). Taken together, our findings suggest that upon acquisition of doxorubicin resistance, autophagy is increased through changes in cellular LC3-II levels and localization, as exhibited by the change from a weak, diffuse, punctate pattern to strong clustered staining in the perinuclear region. Moreover, our observations are consistent with previously described changes in the localization of LC3-II upon induction of autophagy.

### Partial co-localization of Mitotracker™ Fluorescence with LC3-II Immunofluoresence

Autophagosomes in perinuclear region of MCF-7_DOX2–10_ cells not only co-localized with lysosomes, but also partially co-localized with mitochondria. This co-localization was revealed by immunohistochemical staining with the LC3-II antibody after MitoTracker™ labeling. As shown in Fig. 3g, both mitochondria (red) and autophagosomes (green) are evenly distributed throughout the cytoplasm in MCF-7_CC10_ cells, with a small amount of overlapping staining (yellow). In contrast, MitoTracker™ labeling and LC3-II staining in MCF-7_DOX2–10_ cells localized predominantly in the perinuclear region, with strong co-localization of staining (Fig. 3h). Such structures staining positively for both LC3-II antibody and MitoTracker™ are most likely autophagosomes containing mitochondria, suggesting that selection for doxorubicin resistance resulted in the strong induction of mitophagy. This was confirmed by subsequent experiments (see below).

### Clustering of organelle-containing vacuoles in the perinuclear region upon acquisition of doxorubicin resistance

**Fig. 4:**
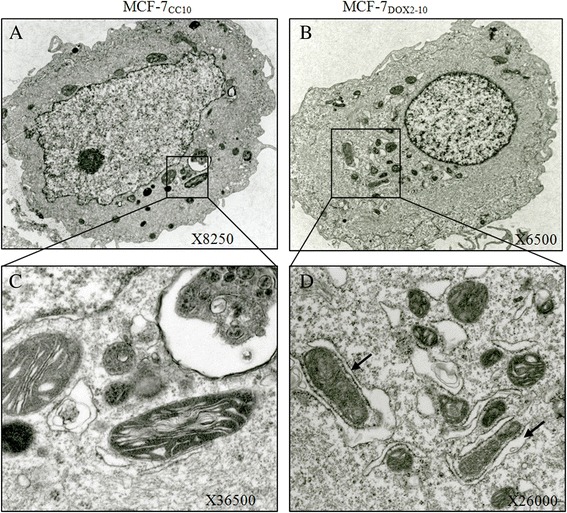
Electron microscopic images of MCF-7_CC10_ and MCF-7_DOX2–10_ cells. Differences in the ultrastructure of MCF-7_CC10_ cells (panel **a**) and MCF-7_DOX2–10_ cells (panel **b**) were visualized by transmission electron microscopy, with Figures **c** and **d** depicting boxed sections in panels (**a** and **b**) at a higher magnification, respectively. Arrows indicate the presence of mitochondria being engulfed by double membrane structures. Five representative images were taken, with 1 image of each cell lines being depicted in the figure

A characteristic property of autophagy is the formation of perinuclear vacuoles called autophagosomes that engulf other organelles [46], which can be visualized by electron microscopy. To further support our hypothesis of autophagy induction upon acquisition of doxorubicin resistance, transmission electron microscopy was used to visualize organelles with high resolution in MCF-7_CC10_ and MCF-7_DOX2–10_ cells. As shown in Fig. 4a and c, electron microscopy images of MCF-7_CC10_ cells revealed that organelles of high electron density (including mitochondria) were well distributed throughout the cytoplasm and were generally not contained within vacuoles (suggesting a lack of organelle autophagy). In contrast, MCF-7_DOX2–10_ cells exhibited numerous cytoplasmic vacuoles in the perinuclear region, some of which contained electron dense organelles. These observations were similar to our findings by confocal microscopy. The presence of abundant organelle-containing vacuoles within the perinuclear region of MCF-7_DOX2–10_ cells (Fig. 4b and d), supports the hypothesis of autophagosome formation upon acquisition of doxorubicin resistance. Some of the electron dense structures in MCF-7_DOX2–10_ cells appear to have cristae reminiscent of mictochondria, and may be late autophagic vacuoles (autophagolysosomes).

### Autophagy as a mechanism for reducing or eliminating organelles damaged by reactive oxygen species in MCF-7_DOX2–10_ cells

A well-studied type of autophagy is selective mitophagy, which mediates cargo-specific removal of damaged mitochondria [47]. Doxorubicin is well known to induce reactive oxygen species (ROS) [29] which results in oxidative damage to both nuclear and mitochondrial DNA [48, 49]. Mitochondria are especially prone to ROS-mediated damage. Our observations using transmission electron microscopy (Fig. 4) and confocal microscopy studies (Fig. 3g and h) did reveal a large increase in the number of cytoplasmic vacuoles in doxorubicin-resistant cells, with a large number of electron dense organelles (likely mitochondria) in or near vacuoles. Thus, selective mitophagy may help doxorubicin-resistant cells rid themselves of damaged mitochondria formed by the continuous exposure to doxorubicin, possibly increasing their survival. Autophagy induction can also neutralize or eliminate the effects of ROS through the Beclin 1-binding protein HMGB1 [50]. To elucidate a possible mechanism for autophagy induction in doxorubicin resistance, the expression of key proteins involved autophagy induction was assessed in MCF-7_CC10_ and MCF-7_DOX2-10_ cells in the presence of bafilomycin. Bafilomycin A1 was added to cells to prevent degradation of proteins through the drug’s ability to block the fusion of autophagosomes with lysosomes as well as the dynamic flux of protein hydrolysis through lysosomes, leading to the accumulation of autophagosomal structures [42].

**Fig. 5:**
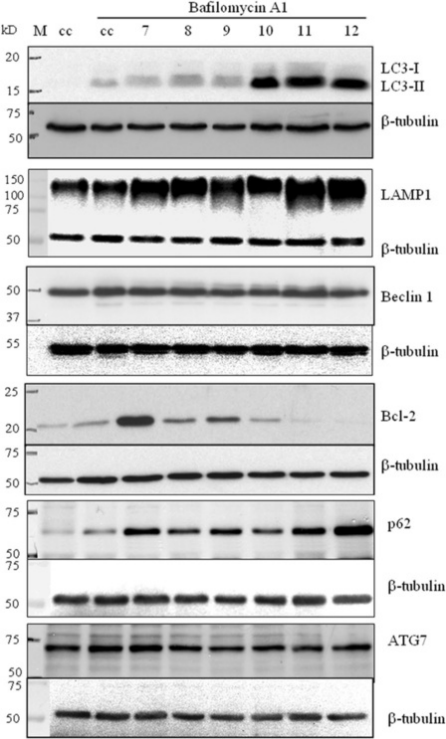
Immunoblots depicting the level of LC3, LAMP1, Beclin 1, Bcl-2, p62 and Atg7 proteins in MCF-7_DOX2_ cells at selection doses 7 through 12 (7–12) grown in the presence of bafilomycin A1. Immunoblots also depict the level of these proteins in MCF-7_CC10_ cells (cc), grown in the absence of bafilomycin A1. Bafilomycin (50 nM) was added to block degradation of the proteins by lysosomal proteases. For control MCF-7_CC10_ cells (cc, lane1), a volume of ethanol equal to that of added bafilomycin A1 was used as a vehicle control. All images are representative of at least three independent experiments. Molecular weight standards are depicted in lane M

Sequestosome 1 (p62) is an ubiquitin-binding adaptor protein, which binds to parkin-ubiquitinated mitochondrial substrates and mediates both the clustering of mitochondria and recruitment of ubiquitylated cargo into autophagosomes by binding to LC3 [51, 52]. In fact, both ubiquitinated protein aggregates and dysfunctional mitochondria are recruited to autophagy machinery through LC3 [52–54]. Consistent with this view, bafilomycin-treated MCF-7_DOX2_ cells exhibited higher levels of p62 than similar-treated MCF-7_CC_ cells (Fig. 5). Moreover, there appeared to be a trend towards increasing p62 expression as doxorubicin selection dose was increased from dose level 7 to dose level 12. Interestingly, the increase in p62 expression (dose 7) preceded the acquisition of doxorubicin resistance at selection dose 9. These findings suggest that selection of breast tumour cells for survival in the presence of doxorubicin results in increasing p62 expression, which helps promote clearance of mitochondria damaged by drug-induced ROS.

### Up-stream proteins involved in the regulation of canonical autophagy appear not to be associated with doxorubicin resistance-related autophagy

The above findings provide several lines of evidence indicating that selection for doxorubicin resistance results in the promotion of autophagy. To begin to explore the mechanisms associated with autophagy induction, the expression of additional key autophagy-related proteins was examined in MCF-7_CC10_ and MCF-7_DOX2_ cells in the presence of bafilomycin. As shown in immunoblotting experiments depicted in Fig. 5, the expression of LAMP1 (a lysosomal protein biomarker indicative of cellular lysosome content) was significantly elevated in MCF-7_DOX2_ cells compared to MCF-7_CC10_ cells, with a trend towards increasing LAMP1 expression with increasing selection dose. In contrast, similar to the β-tubulin loading control, cellular Beclin 1 and Atg7 levels remained unchanged upon selection for doxorubicin resistance. Interestingly, Bcl-2 levels at low selection doses (up to dose 7) were clearly higher than in MCF-7 cc cells at equivalent selection doses. However, Bcl-2 expression then decreased dramatically as selection dose was progressively increased, such that expression was barely detectable in MCF-7_DOX2_ cells at selection dose 12 (Fig. 5). Given that Bcl-2 binds to Beclin 1 to inhibit autophagy [55], this reduction in cellular Bcl-2 levels provides some insight into possible mechanisms by which selection for doxorubicin resistance activates autophagy (see Discussion).

LC3 conjugation to the nascent autophagic vacuolar membrane is required for the initiation of autophagy and the late steps of autophagy after the isolation membrane has formed [44]. This involves conjugation of LC3-I to phosphatidylethanolamine [56], which, in turn, causes a change in LC3 localization from the cytoplasm (LC3-I) to the autophagosomal membrane (LC3-II). As shown in Fig. 5, cellular levels of LC3-II (a well-established biomarker of late autophagy) were very strongly increased when the doxorubicin selection dose was equal to or above dose 10. Interestingly, this corresponded very well with the selection doses where strong levels of doxorubicin resistance were obtained (resistance factors >2-fold; Fig. 1). Moreover, we observed that LC3-I levels were extremely low at the beginning of selection but increased slightly as doxorubicin resistance was achieved. This elevated expression of LC3-II was not due to blocked flux to lysosomes in MCF-7_DOX2–10_ cells because LC3-II protein levels in all samples were assessed in the presence of bafilomycin A1, which blocks the degradation of LC3-II (Fig. 5).

**Fig. 6:**
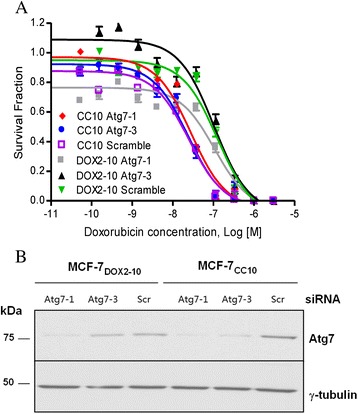
Effect of *ATG7*-specific siRNAs (Atg7-1 or Atg7-3) or a scrambled control siRNA (Scramble) on doxorubicin sensitivity and expression of Atg7 protein in MCF-7_CC10_ and MCF-7_DOX2–10_ cells. Doxorubicin sensitivity was assessed using clonogenic assays (**a**), while the efficiency of gene knockdown was assessed in immunoblotting experiments (**b**) using antibodies specific for the Atg7 protein. A γ-tubulin antibody was used as a loading control

Atg7 plays an important role in late autophagosome formation. We thus assessed whether siRNA-mediated knockdown of ATG7 transcript expression could block autophagy in MCF-7_DOX2–10_ cells in the presence of doxorubicin, thereby restoring doxorubicin sensitivity. As shown in Fig. 6b, a siRNA specific for the *ATG7* transcript (Atg7-1) was able to strongly reduce Atg7 protein expression in both MCF-7_CC10_ and MCF-7_DOX2–10_ cells, as measured in immunoblotting experiments with an Atg7 specific antibody. Another siRNA (Atg7-3) was able, to a lesser extent, to suppress Atg7 expression in MCF-7_CC10_ cells, but had only a small effect on Atg7 expression in MCF-7_DOX2–10_ cells. These siRNAs had no effect on β-tubulin protein expression, nor did a control scrambled Atg7 siRNA sequence have any effect on Atg7 or β-tubulin protein expression. Nevertheless, despite the effects of the Atg7 siRNAs on Atg7 expression, these siRNAs had no significant effect on doxorubicin sensitivity in either MCF-7_CC10_ or MCF-7_DOX2–10_ cells (Fig.6a). Taken together, these and the above findings suggest that changes in Atg7 and Beclin 1 expression did not appear to be associated with the induction of drug resistance in MCF-7_DOX2–10_ cells.

### The canonical autophagy pathway is intact in MCF-7_DOX2–10_ cells but does not appear to be involved in autophagy associated with doxorubicin resistance

**Fig. 7:**
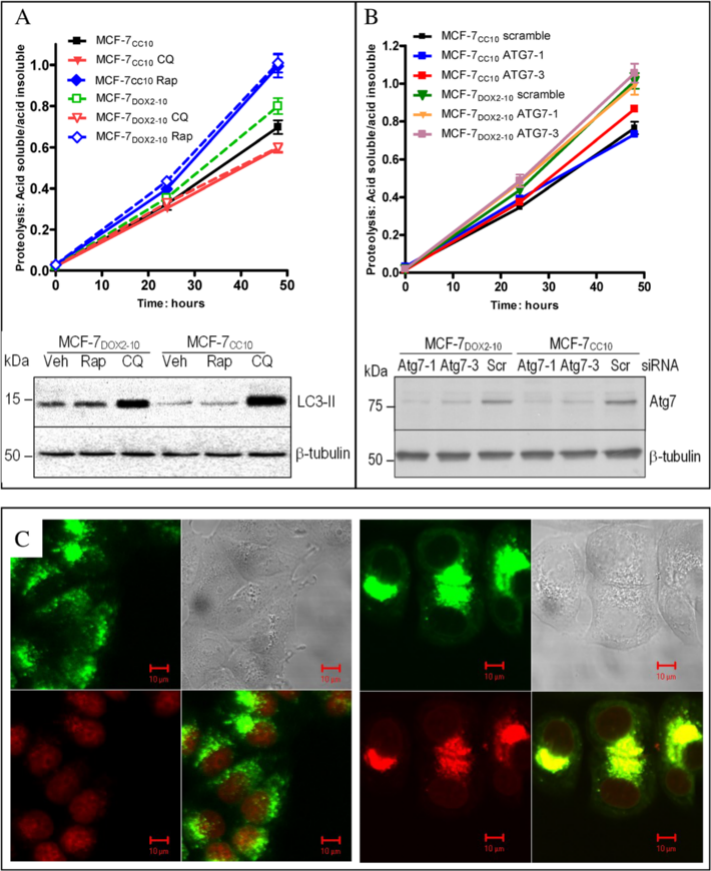
Effect of rapamycin, chloroquine, an *ATG7*-specific siRNA or an scrambled control siRNA on long lived protein turnover (flux assay), LC3-II levels, or lysosome and doxorubicin localization in MCF-7_CC10_ and MCF-7_DOX2–10_ cells. The flux assay was conducted to examine the overall hydrolysis of long lived proteins through autophagy after cells are treated with the autophagy activator rapamycin (Rap), the autophagy inhibitor chloroquine (CQ) (A, upper panel), or siRNAs specific for the *ATG7* gene or a scrambled control (B, upper panel). Immunoblot analysis was used to assess LC3-II protein accumulation in the cells that were treated with either rapamycin (Rap) or chloroquine (CQ) for 24 h compared to a control solution DMSO (vehicle; veh) (**a**, lower panel). The efficiency of Atg7 protein knockdown by siRNA interference (**b**, lower panel) compared to scramble control (scr) was also assessed in this experiment using immunoblot analysis with anti-Atg7 antibodies. Confocal microscopy examination (panel **c**) was also performed to show the effect of chloroquine on the subcellular distribution of lysosomes (green) and doxorubicin (red) in MCF-7_CC10_ cells (**c**, left) and MCF-7_DOX2–10_ cells (**c**, right). MCF-7 cc10 cells were treated with 10 μM of chloroquine and 2 μM of doxorubicin for 8 h, and MCF-7_D__OX2-10_ cells were treated with 10 μM of chloroquine and 2 μM of doxorubicin for 48 h. The images in panel C represent one of the 10 microscopic photos from two sets of separately stained slides in two independent experiments. The staining and phenotype were very consistent throughout 100 viewed cells

We have provided evidence of increased autophagy in MCF-7 cells upon selection for doxorubicin resistance, including changes in the expression and localization of LC3-II. Such cells with increased autophagy would be expected to exhibit higher rates of protein turnover, since autophagy promotes degradation of damaged or defective proteins or cellular organelles. We thus examined the rate of protein turnover in MCF-7_CC10_ and MCF-7_DOX2–10_ cells in a standard flux assay used in the assessment of autophagy. As shown in Fig. 7a, the autophagy activator rapamycin and the autophagy inhibitor chloroquine stimulated and inhibited protein turnover in the flux assay, respectively, in both MCF-7_CC10_ and MCF-7_DOX2–10_ cells. While there was no statistically significant difference in the rates of protein turnover between the MCF-7_CC10_ and MCF-7_DOX2–10_ cells at 24 h. At 48 h, MCF-7_DOX2–10_ cells exhibited higher rates of protein turnover than MCF-7_CC10_ cells (Fig. 7a and b). However, this difference in the flux rates between MCF-7_DOX2–10_ cells and MCF-7_CC10_ cells was not observed in the presence of rapamycin or chloroquine. Taken together, the above findings question whether increased protein turnover through an autophagic process was responsible for the observed resistance to doxorubicin in MCF-7_DOX2–10_ cells, in particular because the rates of protein turnover were only marginally different between MCF-7_DOX2–10_ cells and MCF-7_CC10_ cells. The above findings thus suggest that a functional canonical autophagic pathway is present in both cell lines and that autophagic protein turnover is higher in MCF-7_DOX2–10_ than in MCF-7_CC10_ cells. However, when we examined the effect of these agents on cellular LC3-II levels, we observed that chloroquine significantly increased LC3-II levels in both MCF-7_CC10_ and MCF-7_DOX2–10_ cells (Fig. 7a, lower panel). This was particularly striking in the latter cell line. In repeated experiments, rapamycin was found to reproducibly increase LC3-II levels in MCF-7_CC10_ cells, but the magnitude of increase was generally small and variable. These findings suggest that the increased rate of protein turnover induced by rapamycin in both cell lines may be through an autophagic mechanism not involving mTOR-Beclin 1-Atg7 pathway. Although Atg7 is a critical protein in canonical autophagy, the expression of Atg7 was not changed during selection for doxorubicin resistance. Atg7 siRNA knockdown did not alter the hydrolysis of long lived proteins as shown in the flux assay (Fig. 7b, upper panel), despite the clearly reduced expression of Atg7 protein in cells transfected with the Atg7 siRNAs (Fig. 7b, lower panel). Chloroquine did not affect localization of lysosomes or doxorubicin in MCF-7_CC10_ cells (Fig. 7c) or MCF-7_DOX2–10_ cells (Fig. 7d).

Given all of our experimental findings to date, MCF-7_DOX2–10_ cells appear to exhibit elevated autophagy, based on the clustering and co-localization of lysosomes and organelles in the perinuclear region, increased cytoplasmic vacuoles containing mitochondria and other electron-dense organelles, elevated MDC staining, increased LC3-II production, and increased protein turnover (autophagic flux). Further evidence that autophagy is nevertheless occurring in MCF-7_DOX2–10_ cells comes from our observations that chloroquine (which inhibits autophagy by blocking the fusion of autophagosomes with lysosomes) inhibits protein flux in MCF-7_DOX2–10_ cells (Fig. 7a) and blocks the ability of these cells to resist killing by doxorubicin (Fig. 1). This is despite no change in the localization of doxorubicin in MCF-7_DOX2–10_ cells in the presence of chloroquine (Fig. 7c), even when clustering of lysosomes is observed. Thus, chloroquine does not appear to be increasing doxorubicin cytotoxicity by altering the localization of doxorubicin, but rather through its ability to inhibit autophagy.

## Discussion

While previous studies have suggested a link between autophagy and chemotherapy drug resistance [57–61], a temporal association between the acquisition of chemotherapy resistance and induction of autophagy has yet to be established. Moreover, it is unclear how this relates to drug uptake and drug localization in drug-resistant cells. In this study, we report for the first time that the acquisition of doxorubicin resistance can be temporally correlated with both enhanced drug sequestration into clustered perinuclear lysosomes and enhanced autophagy. The induction of autophagy upon acquisition of drug resistance is associated with increased and decreased cellular p62 and Bcl-2 levels, respectively. Inhibition of autophagy by chloroquine promotes doxorubicin-induced cell death in MCF-7_DOX2–10_ cells, but not in drug-sensitive MCF-7_CC10_ cells.

It has been well established that LC3 is a reliable marker of the formation of autophagosomes in mammalian cells [62]. Its localization within cells changes from a diffuse cytosolic pattern to a punctate pattern representing its recruitment to the autophagosomal membrane during the induction of autophagy [63]. The findings of our study are consistent with this view, since MCF-7 cells selected for survival in increasing concentrations of doxorubicin exhibited increased levels of LC3-II and this increase was temporally associated with acquisition of doxorubicin resistance. Moreover, the location of autophagosomes (LC3-II) and lysosomes (LAMP1) changed upon selection for doxorubicin resistance from a diffuse pattern throughout the cytoplasm to being clustered in the perinuclear region (Fig. 3). Similar to our observations in MCF-7_DOX2_ cells, lysosomal clustering and increased cellular LC3-II levels took place during independent selection of MCF-7 cells for acquired resistance to several other chemotherapy drugs, including an analog of doxorubicin (epirubicin), and both the taxanes paclitaxel and docetaxel. These changes took place at or above selection doses where drug resistance was obtained (data not shown). Taken together, these observations suggest that increased autophagy and/or sequestration of drugs in lysosomes are highly reproducible and common mechanisms through which tumor cells acquire resistance to cytotoxic chemotherapy drugs.

Doxorubicin may have at least four possible fates upon entry into MCF-7_DOX2–10_ cells. In a prior study, we have provided evidence that doxorubicin may be metabolized by cytoplasmic aldo-keto reductases (AKRs) into a considerably less toxic metabolite (13-OH doxorubicinol) in breast tumor cells [64]. Alternatively, the drug may be sequestrated into lysosomes (either as doxorubicin or its 13-OH metabolite), due to its properties as a weak base [21, 65]. Thirdly, before reaching the nucleus, doxorubicin may bind to mitochondrial DNA and induce oxidative damage to mitochondria (due to the drug’s ability to generate ROS). This, in turn, may result in the activation of DNA damage response/survival pathways [66]. Finally, we have previously provided evidence that at higher selection doses, doxorubicin may simply be actively effluxed from MCF-7_DOX2–10_ cells through the induced expression of drug transporters such as Abcc1 [61]. All of these mechanisms may explain why only a small amount of doxorubicin appears to be present in the nuclei of MCF-7_DOX2–10_ cells (Fig. 2).

During selection for doxorubicin resistance, it would be expected that doxorubicin would bind to mitochondrial DNA, thereby exposing the organelles to ROS produced by doxorubicin [66]. This may result in large numbers of damaged mitochondria, which would be targeted for degradation by activation of a particular form of autophagy (namely mitophagy). This would be consistent with observations of many vesicularized mitochondria in MCF-7_DOX2–10_ cells (Fig. 4). In addition, activation of autophagy has been reported to increase cellular capacity to survive stress associated with exposure to ROS [67]. Since canonical autophagy requires the involvement of all Atg proteins [68] and since knockdown of Atg7 did not significantly reduce doxorubicin resistance, this suggests that acquisition of doxorubicin resistance may be associated with the induction of non-canonical autophagy [9]. The mechanism for autophagy associated with selection for doxorubicin resistance may involve selective delivery of damaged organelles into autophagosomes that then fuse with lysosomes for hydrolytic degradation [69, 70], even under nutrient-rich conditions. This form of non-canonical autophagy is often referred to as selective autophagy.

It has been suggested that p62, as a selective cargo receptor, is involved in linking ubiquitinated protein aggregates to the autophagy machinery through LC3 [52, 54]. In addition, p62 mediates the clustering and aggregation of dysfunctional mitochondria and binds to LC3-II to deliver aggregated mitochondria to autophagosomes [53]. Increased p62 expression upon selection for survival in increasing concentrations of doxorubicin (beginning at selection dose 7) would help facilitate this delivery of dysfunctional mitochondria to autophagosomes. While Atg7 and Beclin1 levels remained unchanged, Bcl-2 protein levels varied throughout selection for doxorubicin resistance (Fig. 5). For example, relative to co-cultured MCF-7_CC_ cells, MCF-7_DOX2_ cells selected to dose level 7 (6.5 nM doxorubicin) showed considerably higher expression of Bcl-2. This increase in cellular Bcl-2 levels likely enabled MCF-7_DOX2_ cells to survive doxorubicin concentrations up to dose level 7, due to the ability of Bcl-2 to inhibit doxorubicin-induced apoptosis [63, 71, 72]. However, at selection doses above 6.5 nM doxorubicin, Bcl-2 expression began to decline in a dose-dependent manner (Fig. 5). Since, Bcl-2 can negatively regulate autophagy by forming complexes with Beclin 1 [55, 73], the loss of Bcl-2 might help promote autophagy at higher selection doses by promoting Beclin 1-dependent autophagy. There was, however, no change in the expression of Beclin 1 and Atg7 throughout selection for doxorubicin resistance, which is often seen in canonical autophagy. This suggests the activation of non-canonical or selective autophagy. There is some recent evidence that, in addition to canonical autophagy, Bcl-2 can regulate non-canonical autophagy, since knockdown of Bcl-2 activity by the Bcl-2 inhibitor Z18 induces autophagy that is unaffected by Beclin 1 and phosphatidyl inositol 3-kinase inhibition [74]. However, overexpression of Bcl-2 in MCF-7_DOX2–10_ cells did not result in autophagy inhibition (as determined by LC3-II expression levels), nor did it increase cellular sensitivity to doxorubicin (data not shown).

Our data clearly illustrates that MCF-7_DOX2–10_ cells demonstrated a higher level of autophagy (as measured by LC3-II expression and electron microscopy) than equivalent co-cultured control cells. However, the rate of long lived protein hydrolysis as measured by the flux assay (a functional indicator of autophagy) was only marginally higher in MCF-7_DOX2–10_ cells than in MCF-7_CC10_ cells (Fig. 7). This may be because the high level of protein hydrolysis seen in canonical autophagy is used to either degrade long lived proteins for housekeeping purposes or energy production under starvation conditions. However, when cells undergo treatment with chemotherapy drugs, there is no shortage of nutrients and growth factors. Thus, organelle damage might be the main effect of drug treatment, and it may be preferable for cells in such instances to activate selective autophagy to eliminate damaged organelles rather than activation of canonical autophagy and protein hydrolysis to support cellular metabolism. After drug entry into tumor cells, mitochondria may be the first target to be affected by doxorubicin prior to its binding to nuclear DNA. Therefore, doxorubicin resistance could be partially attributed to enhanced clearance of the damaged mitochondria caused by doxorubicin via mitophagy.

Autophagy is a process that receives inputs from multiple pathways. The well documented canonical pathways regulating starvation-induced autophagy [75–77] may or may not be applicable to autophagy induced by other stress inducers, such as chemotherapy agents. For example, the neurotoxin MMP+ induces autophagy in SHSY5Y human neuroblastoma cells through a pathway distinct from starvation-induced autophagy. Classic inhibitors of amino acid deprivation-associated autophagy do not inhibit the autophagic response elicited by MMP+ treatment, despite confirmation that the pathway is operative in SHSY5Y cells [10]. Similarly, MCF-7 cells show Beclin 1-hVps34-independent autophagy or non-canonical autophagy in response to resveratrol treatment [9, 78].

In a recent study, Sun et al. provided evidence of increased autophagy upon exposure of MCF-7 cells to epirubicin and that autophagy facilitates resistance to epirubicin [59]. Our manuscript supports the general themes of the prior study, but differs from it in several respects. Firstly, our study demonstrates a clear dose-dependent and temporal relationship between doxorubicin selection dose and both the acquisition of doxorubicin resistance and increased autophagy, in particular at selection doses at or above 44 nM doxorubicin. We show much greater LC3-II production (autophagy) than that observed by Sun et al. when the selection dose reaches 44 nM or greater. Our study also provides evidence that autophagy induction upon selection for doxorubicin resistance appears unrelated to starvation-induced (canonical) autophagy, as siRNA-mediated downregulation of Atg7 had no effect on the sensitivity of MCF-7_DOX2–10_ cells to doxorubicin and induction of the cargo protein p62 is typically associated with non-canonical or selective autophagy.

There is emerging evidence that autophagy may be highly relevant to chemotherapy drug resistance and improving the efficacy of chemotherapy treatment in cancer patients. For example, the combined inhibition of autophagy by the mTOR inhibitor temsirolimus and by the lysosomotropic agent chloroquine in a phase I study, showed the combination to be safe, with clear evidence of autophagy inhibition. 67 % of patients achieved stable disease at the maximally tolerated dose (MTD) of this regimen in patients with solid tumours. Moreover, 74 % of melanoma patients achieved stable disease at the MTD of this regimen [79]. The combination of an mTOR and autophagy inhibitor may be important for clinical efficacy, as a study in prostate tumour xenograft models found that the combination of the mTOR inhibitor AZD5363 and chloroquine significantly reduced tumour volume, while either drug alone did not [80]. Further evidence of the potential link between autophagy and response to chemotherapy stems from a phase II study on the efficacy of sorafenib in patients with refractory lymphoma. Patients clinically responsive to sorafenib had higher baseline levels of an autophagic biomarker and experienced a significant reduction in this biomarker during treatment [81]. These previous investigations and our current study in drug-resistant breast tumour cells provide a compelling rationale for investigating the potential of autophagy inhibitors (possibly in combination with mTOR inhibitors) to improve clinical response to chemotherapy. This is particularly important for invasive breast cancer (not including ductal carcinoma in situ), which affects approximately 1 in 8 women in the U.S. (http://www.breastcancer.org/symptoms/understand_bc/statistics ). According to the ClinicalTrials.gov website, two phase II clinical trials are currently recruiting patients to assess the effect of the lysosomotropic autophagy inhibitor chloroquine (alone) in patients with breast cancer or ductal carcinoma in situ prior to surgery.

## Conclusion

This study provides new insight into the multiple mechanisms involved in acquired doxorubicin resistance in breast tumour cells.  In addition to previously the known mechanism involving the increased production of the Abcc1 drug efflux transporter, the cells acquire doxorubicin resistance by sequestering the drug into lysosomes and by activating non-canonical autophagy through increased production of LC3-II and p62.

## Abbreviations

CQ: Chloroquine
LC3: Microtubule-associated protein 1 light chain 3
MCF-7cc: Co-cultured MCF-7 cells
MCF-7_DOX2_: Doxorubicin resistant MCF-7 cells
MDC: Monodansylcadaverine
Rap: Rapamycin
Scr: Scramble siRNA
